# AdImpute: An Imputation Method for Single-Cell RNA-Seq Data Based on Semi-Supervised Autoencoders

**DOI:** 10.3389/fgene.2021.739677

**Published:** 2021-09-08

**Authors:** Li Xu, Yin Xu, Tong Xue, Xinyu Zhang, Jin Li

**Affiliations:** ^1^College of Computer Science and Technology, Harbin Engineering University, Harbin, China; ^2^Key Laboratory of Symbolic Computation and Knowledge Engineering of Ministry of Education, Jilin University, Changchun, China; ^3^School of Mathematics, Harbin Institute of Technology, Harbin, China

**Keywords:** scRNA-seq, missing value filling, semi-supervised learning, autoencoder, imputation method

## Abstract

**Motivation:** The emergence of single-cell RNA sequencing (scRNA-seq) technology has paved the way for measuring RNA levels at single-cell resolution to study precise biological functions. However, the presence of a large number of missing values in its data will affect downstream analysis. This paper presents AdImpute: an imputation method based on semi-supervised autoencoders. The method uses another imputation method (DrImpute is used as an example) to fill the results as imputation weights of the autoencoder, and applies the cost function with imputation weights to learn the latent information in the data to achieve more accurate imputation.

**Results:** As shown in clustering experiments with the simulated data sets and the real data sets, AdImpute is more accurate than other four publicly available scRNA-seq imputation methods, and minimally modifies the biologically silent genes. Overall, AdImpute is an accurate and robust imputation method.

## Introduction

With the development of high-throughput sequencing technology, the emergence of single-cell RNA sequencing (scRNA-seq) technology in genomic sequencing has become a hot topic in recent years (Wagner et al., 2016; Kalisky et al., 2018). Compared with bulk RNA sequencing sequences, scRNA sequences have a relatively high noise level, especially due to so-called dropouts (Vallejos et al., 2015; Lun et al., 2016; Ziegenhain et al., 2017). Dropouts are a special type of missing values due to low RNA input in sequencing experiments and the randomness of gene expression patterns at the single cell level. The presence of dropouts often misleads downstream analysis, such as data visualization, cell clustering, and differential expression analysis (Stegle et al., 2015; Bacher and Kendziorski, 2016; Svensson et al., 2017).

Based on different principles, a variety of single cell RNA-seq data imputation methods have been proposed (Chen and Zhou, 2018; Huang et al., 2018; Van Dijk et al., 2018; Eraslan et al., 2019; Hu et al., 2020; Qi et al., 2021). ScImpute (Li and Li, 2018) divides genes into two groups based on dropout probability (unreliable and reliable classification: *A*_*j*_, *B*_*j*_), and the dropout probability is estimated by a mixed model. Scientific computing estimates *A*_*j*_ by processing *B*_*j*_ as gold standard data. In the first version, a weighted lasso model is used to find similar cells among other cells in *B*_*j*_ genes. Then use the linear regression model of the most similar unit as the estimate of *A*_*j*_. DrImpute (Gong et al., 2018) is an integrated method, which is designed based on the consistent clustering results of scRNA-seq data. In other words, it performs multiple clusters and imputes based on the average of similar cell expression. AutoImpute (Talwar et al., 2018) is a method of imputing dropouts based on an autoencoder. It uses over-complete autoencoders to capture the distribution of given sparse gene expression data, and regenerates complete expression data. DeepImpute (Zhang and Zhang, 2020) is an imputation method based on deep neural networks. The method uses missing layers and loss functions to learn patterns in the data to achieve accurate imputation.

**FIGURE 1:**
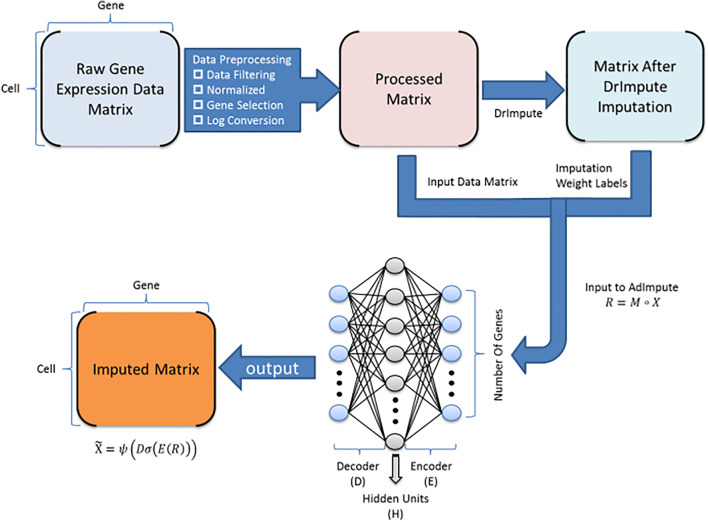
AdImpute pipeline: the pre-processing stage of AdImpute requires screening of raw gene expression data, normalizing by library size, and pruning through gene selection and logarithmic transformation. Afterward, AdImpute first fills the pre-processed matrix with DrImpute, and uses the result of DrImpute as an imputation weight label. Then the label is input into the AdImpute model together with the pre-processed matrix to learn gene expression data. Finally, the missing data value filling and the input matrix reconstruction are done.

At present, machine learning methods are increasingly used in bioinformatics, and many achievements have been made (Peng et al., 2021a, b). We have conducted a lot of clustering experiments on the existing imputation method. According to the experimental results, we found that the machine learning methods did not perform well. The analysis revealed two reasons. One is a large number of zeros in the raw data, making it difficult for machine learning methods to extract deep information from the data. Instead, most of the zeros are regarded as true zeros, that is, no padding is performed, so the data filled by the machine learning method is more discrete. The second reason is that after using some deep learning-based missing value filling methods to fill in, the output data contains negative values, but the actual gene expression values should all be non-negative values. Based on this, we propose an imputation method AdImpute (Figure 1) based on a semi-supervised autoencoder, which combines ordinary imputation methods with machine learning methods to better implement imputation.

An Autoencoder is a type of artificial neural network used in semi-supervised learning and unsupervised learning. Its function is to perform representation learning on the input information by using the input information as the learning target. A number of recent studies describe applications of autoencoders in molecular biology.

In order to solve the problem of difficult to extract the deep information of the data, AdImpute introduces a set of data imputed by DrImpute as an imputed weight label (DrImpute method can be replaced, this article selects the current mainstream method, if there is a better one, you can replace it). While using the autoencoder to impute dropouts, AdImpute adds an imputation weight term to the cost function and compares it with the label data. For a zero value that may be a missing value, the larger the label data value is, the more likely it is to be a missing value, so as to achieve semi-supervised learning. We also give a Relu activation function to the decoding layer to solve the situation of negative values in the filled data.

An example is given to better understand the principle of this method. If we compare the imputation process to an exam, then the unsupervised machine learning method is to complete a test paper normally, and supervised machine learning is to complete a test paper under the premise of having a standard answer. Semi-supervision is equivalent to finding a test paper of a student with good grades as a reference to complete my test paper.

In reality, supervision is meaningless for imputation. Current machine learning algorithms are all based on unsupervised. Here, we first proposed the idea of applying semi-supervision to imputation, and verified the superiority with the help of clustering results.

## Materials and Methods

### Autoencoder

**FIGURE 2:**
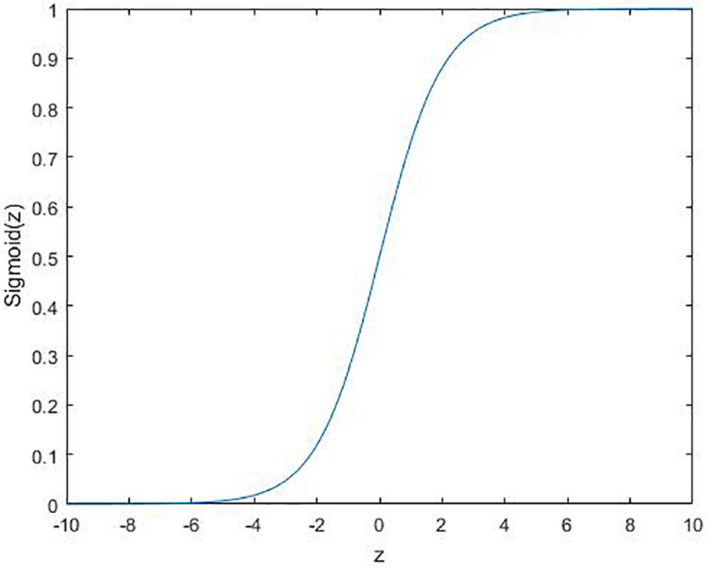
Sigmoid function: $f\left(z\right)=\frac{1}{1+e^{-z}}$.

**FIGURE 3:**
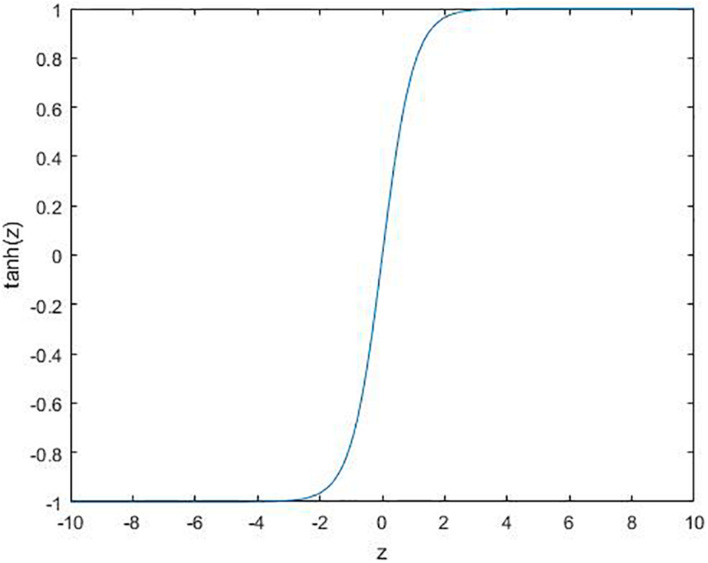
Tanh function: $\text{tanh}\left(z\right)=\frac{e^{z}-e^{-z}}{e^{z}+e^{-z}}$.

**FIGURE 4:**
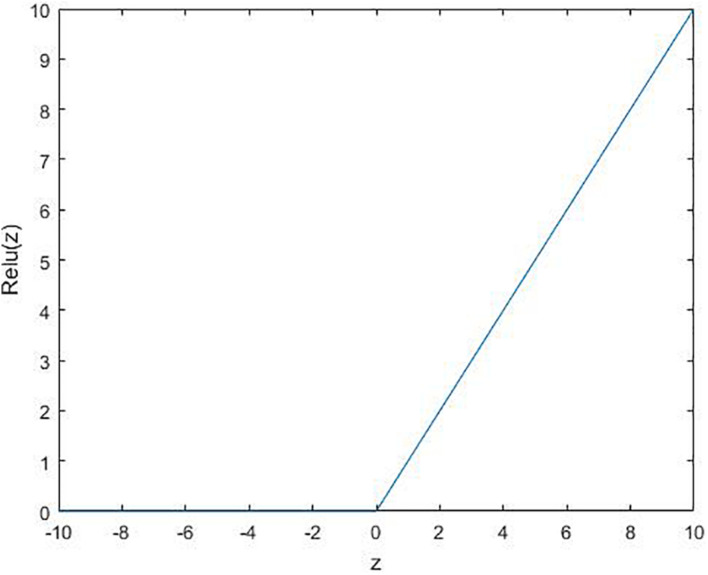
Relu function: relu(*z*) = max⁡(0, *z*).

In simple terms, the autoencoder is the process of reducing the dimension after encoding the raw data, so as to discover the hidden rules among the data. The autoencoder is composed of encoder *E* and decoder *D*. The encoder first maps the input data *X* to the latent space *H*:

(1) H=ϕ(EX)

where ϕ is the activation function. Several commonly used functions are shown in Figures 2–4.

In the training phase, the encoder and decoder are usually learned by minimizing the Euclidean cost function:

(2) $$\arg\;\min_{D,E}{||\text{X – D}\phi \left(\mathrm{EX}\right)||}_{F}^{2}$$

There are several variants of the autoencoder model: multi-layer autoencoder and regularized autoencoder. The multi-layer autoencoder is created by nesting the autoencoder inside another autoencoder. Mathematically, this is expressed as:

(3) $$\arg\min_{D^{\prime }s,E^{\prime }s}||{\text{X – D}}_{1}\phi (D_{2}\cdots \phi D_{N}(\phi \left(E_{N}\cdots \phi E_{1}\left(X\right)\cdots \right)|{|}_{F}^{2}$$

The cost function used by the regularized autoencoder can encourage the model to learn other features, rather than copying the input to the output. These characteristics include sparse representation, robustness to noise or missing inputs, etc. Even if the model capacity is large enough to learn a meaningless identity function, the nonlinear and over-complete regular autoencoder can still learn some useful information about the data distribution from the data. The regularized autoencoder can be expressed as follows:

(4) $$\arg\;\min_{D,E}{||\text{X – D}\phi \left(EX\right)||}_{F}^{2}+\lambda \text{ℜ}\left(E,D\right)$$

where λ is the regularization coefficient, and the regularizer R is a real function about *E* and *D*.

### The Design and Implementation of AdImpute

AdImpute is a missing value filling method based on semi-supervised autoencoder. While using a complete autoencoder to capture the distribution of the given sparse gene expression data, AdImpute introduces the data filled by DrImpute as the imputation weight label of the model, which makes the regenerated complete expression data obtain higher quality.

The purpose of AdImpute is to estimate these dropouts by looking for the full version of gene expression data. The model of the measured value is:

(5) R=M∘X

where ° is the Hadamard product, *M* is a binary mask containing 1, *R* contains a non-zero term, and elsewhere is 0. *X* represents the count matrix to be estimated.

AdImpute needs to import the data filled by DrImpute into the model as the imputation weight label of the model, which is recorded as *F*. Then the sparse gene expression matrix *M*°*X* is input into the autoencoder, and it is trained to learn the best encoder and decoder functions by minimizing the cost function. In order to prevent the overfitting of non-zero values in the count matrix, we regularize the learned encoder and decoder matrices. The cost function is as follows:

(6) $$\begin{array}{c} \min_{D,E}{||\text{R – D}\sigma \left(E\left(R\right)\right)||}_{O}^{2}+\delta {||\text{F-D}\sigma \left(E\left(R\right)\right)||}_{O}^{2}\\ +\frac{\lambda }{2}\delta \left({||E||}_{F}^{2}+{||D||}_{F}^{2}\right) \end{array}$$

where *E* is the encoder matrix, *D* is the decoder matrix, and λ is the regularization coefficient. In the formula (6), $\delta {||\text{F – D}\sigma \left(E\left(R\right)\right)||}_{O}^{2}$ is the imputation weight item, *F* is the imputation weight label, and δ is the weight of the imputation weight item. ||⋅||_*O*_ means that the loss is calculated only for the non-zero counts present in the sparse expression matrix *M*∘*X*, and σ is the Sigmoid activation function applied to the encoder layer in the neural network.

Finally, after the training and learning encoder and decoder matrix, the filled expression matrix is as follows:

(7) $$\tilde{\text{X}}=\psi \left(D\sigma \left(E\left(R\right)\right)\right)$$

where ψ is the Relu activation function applied to the decoder layer in the neural network.

The AdImpute model consists of a fully connected multi-layer perceptron with three layers: input layer, hidden layer and output layer. The model uses an imputation weight label composed of DrImpute-filled data to improve the missing value filling effect. The gradient is calculated by back propagation of the error, and the gradient descent method is used for training to reach the minimum value of the cost function (6). The RMSProp Optimizer is used to adjust the learning rate so as to avoid falling into a local minimum and reach the minimum of the cost function faster. Both the encoder matrix *E* and the decoder matrix *D* are subject to the initialization of random normal distribution. The output of the decoder uses Relu as the activation function.

The selection of hyper-parameters is as follows:

(1) Regularization coefficient λ is used to control the contribution of the regular term to the cost function.
(2) The weight δ of the imputation weight term is used to control the contribution of the imputation weight term to the cost function.
(3) The size of the hidden layer (the dimension of the potential space).
(4) The initial value of the learning rate.
(5) Threshold. The change of the cost function value in successive iterations is less than the threshold value, which means convergence and stops the gradient descent.

## Results

A good imputation method can retain most of the real available information for the raw data. Therefore, in order to measure the quality of the missing value filling methods, we choose cluster analysis in the downstream analysis. We will select some data sets and use five methods to impute the dropouts, and use the results to perform cluster analysis.

By analyzing the results of the clustering, we estimated the advantages and disadvantages of the dropouts imputation methods. The cluster evaluation indicators used in this paper are rand, ARI, FM, and Jaccard.

### The Clustering Experiment on the Simulated Data Sets

**FIGURE 5:**
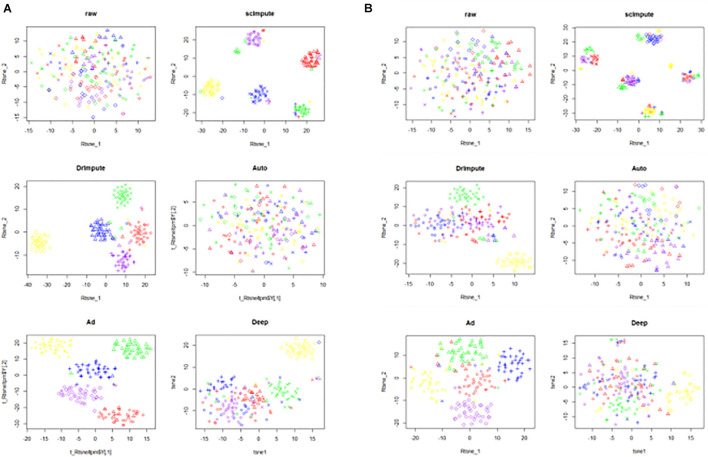
The visualization results of clustering on the simulated data sets. The six images are the clustering results of the raw data and the clustering results imputed by scImpute, DrImpute, AutoImpute, AdImpute, and DeepImpute. And **(A)** is the clustering visualization results on simu1 data set; **(B)** is the clustering visualization results on simu2 data set.

We use CIDR (Lin et al., 2017) to generate two simulated data sets simu1 and simu2. The details of simu1 and simu2 is provided in the section “Data availability.” We label the generated data and mark the actual cell clustering results. After preprocessing the raw data, we use scImpute, DrImpute, AutoImpute, AdImpute, and DeepImpute to impute the dropouts. Based on the imputed data results, T-SNE for dimensionality reduction and visualization is carried out, and then K-means clustering is used. The visualization results of clustering are shown in Figure 5.

**FIGURE 6:**
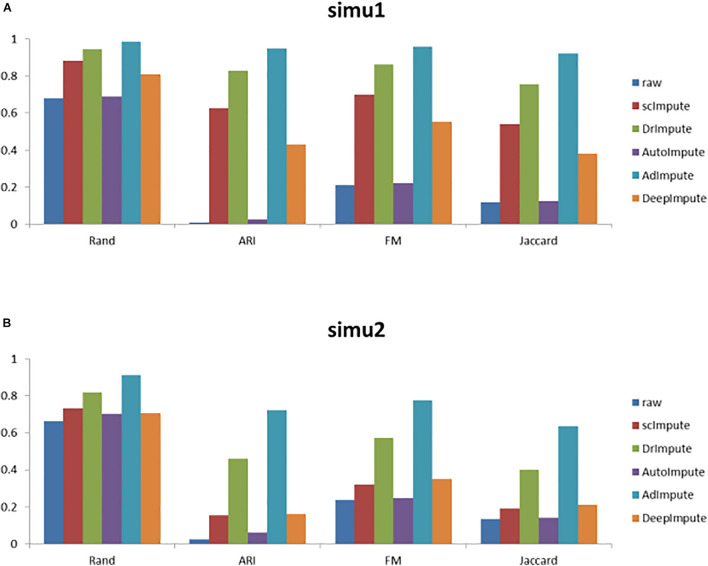
The histogram of clustering evaluation indexes on the simulated data sets, including the clustering evaluation indexes of raw data and the clustering evaluation indexes after imputing by scImpute, DrImpute, AutoImpute, AdImpute, and DeepImpute. And **(A)** is the clustering evaluation indexes on simu1 data set; **(B)** is the clustering evaluation indexes on simu2 data set.

Based on the clustering results, we calculate the cluster evaluation indexes. The results are given by Supplementary Table 1. In order to analyze the experimental results more intuitively, we give a histogram of clustering evaluation indexes in Figure 6.

Analyzing the results of the above experiments, we can find that AdImpute has a very good performance in the clustering experiment on the simulated data sets. The performance of AutoImpute is not ideal, scImpute and DeepImpute are always slightly inferior than DrImpute. In general, AdImpute performs best on the simulated data sets. And the ranking of clustering effects is shown in Table 1.

**TABLE 1:** The ranking of clustering effects on the simulated data sets.

	scImpute	DrImpute	AutoImpute	AdImpute	DeepImpute
simu1	3	2	5	1	4
simu2	4	2	5	1	3

*1 represents the best and 5 represents the worst in the table.*

### The Clustering Experiment on the Real Data Sets

**FIGURE 7:**
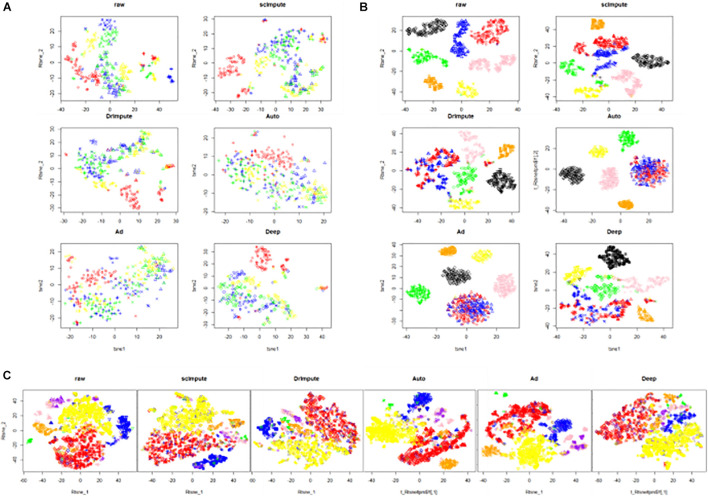
The visualization results of clustering on the real data sets. The six images are the clustering results of the raw data and the clustering results imputed by scImpute, DrImpute, AutoImpute, AdImpute, and DeepImpute. And **(A)** is the clustering visualization results on Trapnell (GSE52529) data set; **(B)** is the clustering visualization results on hPSC (GSE75748) data set; **(C)** is the clustering visualization results on Romanov (GSE74672) data set.

In the part, we select three real data sets: Trapnell (Trapnell et al., 2014), hPSC (Chu et al., 2016), and Romanov (Romanov et al., 2017). After preprocessing the raw data, we use scImpute, DrImpute, AutoImpute, AdImpute, and DeepImpute to impute the dropouts. Based on the imputed data results, T-SNE for dimensionality reduction and visualization is carried out, and then K-means clustering is used. The visualization results of clustering are shown in Figure 7.

**FIGURE 8:**
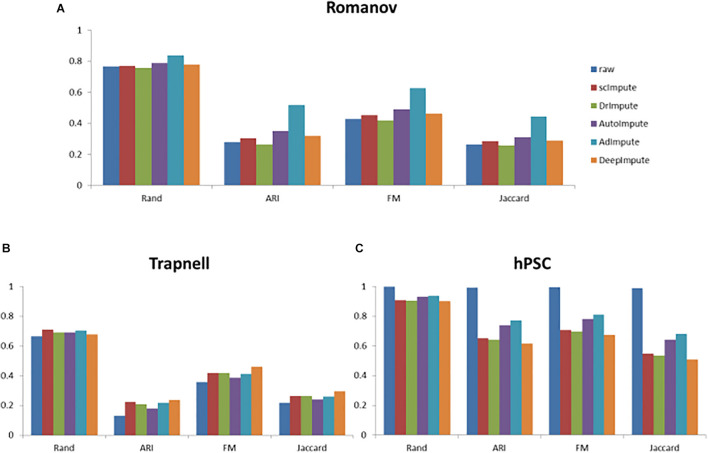
The histogram of clustering evaluation indexes on the real data sets, including the clustering evaluation indexes of raw data and the clustering evaluation indexes after imputing by scImpute, DrImpute, AutoImpute, AdImpute, and DeepImpute. And **(A)** is the clustering evaluation indexes on Trapnell (GSE52529) data set; **(B)** is the clustering evaluation indexes on hPSC (GSE75748) data set; **(C)** is the clustering evaluation indexes on Romanov (GSE74672) data set.

Based on the clustering results, we also calculate the cluster evaluation indexes. The results are given by Supplementary Table 2. In order to analyze the experimental results more intuitively, we give a histogram of clustering evaluation indexes in Figure 8.

Analyzing the experimental results, we can find that AdImpute still has a good performance in the clustering experiment on the real data sets. Despite being slightly inferior to scImpute in Trapnell data set, the overall performance is still the best among these methods. AutoImpute and DeepImpute do not perform well on the simulated data sets, but behave well on the real data sets. The performance of scImpute is unstable, and DrImpute is not ideal. Through the results on hPSC data set, we can see that AdImpute has minimally modified the expression of real biological silencing genes. Overall, AdImpute still performs best on the real data sets. And the ranking of clustering effects is shown in Table 2.

**TABLE 2:** The ranking of clustering effects on the real data sets.

	scImpute	DrImpute	AutoImpute	AdImpute	DeepImpute
Trapnell	1	4	5	2	3
hPSC	3	4	2	1	5
Romanov	4	5	2	1	3

*1 represents the best and 5 represents the worst in the table.*

## Discussion

Since the scRNA-seq data suffers from dropout events that hinder the downstream analysis of data, we propose a statistical imputation method AdImpute to denoise the scRNA-seq data. AdImpute aims to implement data recovery and maintain the heterogeneity of gene expression across cells. One of the advantages of AdImpute is that it can be incorporated into most of the downstream analysis tools for the scRNA-seq data. In this paper, we perform downstream analysis experiments in the simulated datasets and real datasets, and the results show that our method improves the raw data and outperforms the other imputation methods.

Rand, ARI, FM, and Jaccard Index were used to measure the clustering results of imputed data. AdImpute performs well in the clustering experiments of the simulated data sets and the real data sets. In the simulated data sets, it can be seen from Figure 6 that the clustering results of AdImpute is significantly better than that of the other three algorithms when *v* = 9/10.

Because the data loss degree of real data is unknown, there may be a large number of true zeros, which can reflect the judgment ability of each algorithm to distinguish between missing zeros and true zeros. The sequencing data in the third data set hPSC has almost no zeros caused by data loss, which can better reflect the judgment ability of the five algorithms. As can be seen from Figure 8, in the Trapnell and Romanov data sets, the clustering effects of the five algorithms after missing value filling are not significantly different. After filled by scImpute, DrImpute, AdImpute, AutoImpute, and DeepImpute, the clustering results are improved compared with the raw data. However, from the experimental results of hPSC data set, we can see that the effect of AdImpute is significantly higher than the other four methods, which shows that AdImpute algorithm has good performance in identifying true zeros. In general, AdImpute performs best on the real data sets.

By comprehensively analyzing the results of the simulated data sets and the real data sets, we draw the following conclusions. scImpute prefers to regard the identified zeros as true zeros, so it performs well on the real data sets, but it does not perform well on the simulated data sets. DrImpute prefers to treat the identified zeros as the missing zeros, so it performs well in the simulated data sets, but it does not perform well in the real data sets. One of the limitations of DrImpute is that it considers only cell-level correlation using a simple hot deck approach. The performance of AutoImpute is not satisfactory on both the simulated data sets and the real data sets, but its effect on hPSC data set is better than that of scImpute, DrImpute, and DeepImpute. AutoImpute behaves ideally in retaining the most of true zeros present in the data. It is speculated that AutoImpute has a poor judgment ability to determine missing values, and most of the identified zeros are considered as true zeros. DeepImpute performs ordinarily on both the simulated data sets and the real data sets. It is designed for the bulk-RNAseq data and is suitable for handling large datasets. Its training and the prediction processes are separate, and DeepImpute tends to fail when the data show large heterogeneity and sparsity, which are two key characteristics of scRNA-seq data. AdImpute has minimally modified the expression of real biological silencing genes, and the filling effect is very robust.

## Data Availability Statement

Publicly available datasets were analyzed in this study. This data can be found here: Trapnell: Primary human myoblast scRNA-seq data, available in the GEO database, accession number GSE52529. hPSC: Human pluripotent stem cell scRNA-seq data, which can be obtained from the GEO database under the accession number GSE75748. Romanov: Mouse hypothalamus scRNA-seq data, which can be obtained in the GEO database under the accession number GSE74672.

## Author Contributions

JL provided the guidance during the whole research. YX and TX collected the data. LX, YX, TX, and XZ carried out the data analysis. YX and LX wrote the manuscript. All authors contributed to the article and approved the submitted version.

## Conflict of Interest

The authors declare that the research was conducted in the absence of any commercial or financial relationships that could be construed as a potential conflict of interest.

## Publisher’s Note

All claims expressed in this article are solely those of the authors and do not necessarily represent those of their affiliated organizations, or those of the publisher, the editors and the reviewers. Any product that may be evaluated in this article, or claim that may be made by its manufacturer, is not guaranteed or endorsed by the publisher.
