# Supplementary material for: AdImpute: An Imputation Method for Single-Cell RNA-Seq Data Based on Semi-Supervised Autoencoders
Source: Front Genet. 2021 Sep 8;12:739677. doi: 10.3389/fgene.2021.739677 (PMC8456123; doi:10.3389/fgene.2021.739677)
Supplement: Supplementary file 1 [file Table_1.DOCX]

| **Table S1.** The results on evaluation indexes of clustering of the simulated data sets. | | | |
| --- | --- | --- | --- |
| **Data set name** | | **simu1** | **simu2** |
| raw | Rand | 0.678141 | 0.662965 |
|  | ARI | 0.008584 | 0.024456 |
|  | FM | 0.210486 | 0.238762 |
|  | Jaccard | 0.117525 | 0.134581 |
| scImpute | Rand | 0.881859 | 0.731508 |
|  | ARI | 0.627469 | 0.152877 |
|  | FM | 0.701105 | 0.320152 |
|  | Jaccard | 0.539742 | 0.190577 |
| DrImpute | Rand | 0.945528 | 0.820352 |
|  | ARI | 0.827885 | 0.459951 |
|  | FM | 0.861819 | 0.574379 |
|  | Jaccard | 0.757169 | 0.401574 |
| AutoImpute | Rand | 0.689095 | 0.701508 |
|  | ARI | 0.025078 | 0.061030 |
|  | FM | 0.219135 | 0.247164 |
|  | Jaccard | 0.123033 | 0.140998 |
| AdImpute | Rand | 0.984372 | 0.912312 |
|  | ARI | 0.950414 | 0.723173 |
|  | FM | 0.960133 | 0.777810 |
|  | Jaccard | 0.923324 | 0.636383 |
| DeepImpute | Rand | 0.808141 | 0.705578 |
|  | ARI | 0.431544 | 0.161007 |
|  | FM | 0.554553 | 0.349889 |
|  | Jaccard | 0.381700 | 0.209845 |

*The data is corresponding to Figure 6.*
