# Supplementary material for: AdImpute: An Imputation Method for Single-Cell RNA-Seq Data Based on Semi-Supervised Autoencoders
Source: Front Genet. 2021 Sep 8;12:739677. doi: 10.3389/fgene.2021.739677 (PMC8456123; doi:10.3389/fgene.2021.739677)
Supplement: Supplementary file 2 [file Table_2.DOCX]

| **Table S2**. The results on evaluation indexes of clustering of the real data sets. | | | | |
| --- | --- | --- | --- | --- |
| **Data set name** | | **Trapnell** | **hPSC** | **Romanov** |
| raw | Rand | 0.6662754 | 0.9983483 | 0.7659831 |
|  | ARI | 0.1305041 | 0.9936879 | 0.2798623 |
|  | FM | 0.3554306 | 0.994665 | 0.4284015 |
|  | Jaccard | 0.2159273 | 0.9893865 | 0.2620299 |
| scImpute | Rand | 0.7101556 | 0.9073704 | 0.7681995 |
|  | ARI | 0.2242562 | 0.6529093 | 0.3033953 |
|  | FM | 0.4171354 | 0.7081195 | 0.451507 |
|  | Jaccard | 0.2635319 | 0.5478846 | 0.2840696 |
| DrImpute | Rand | 0.6915051 | 0.9060993 | 0.7577478 |
|  | ARI | 0.2089498 | 0.6421908 | 0.2648398 |
|  | FM | 0.4185021 | 0.6977806 | 0.4182029 |
|  | Jaccard | 0.2640022 | 0.5358333 | 0.256383 |
| AutoImpute | Rand | 0.6898386 | 0.9315584 | 0.7883152 |
|  | ARI | 0.1789678 | 0.7408508 | 0.3512653 |
|  | FM | 0.3864829 | 0.7814694 | 0.4885584 |
|  | Jaccard | 0.2394912 | 0.6412507 | 0.3111056 |
| AdImpute | Rand | 0.705301 | 0.9387679 | 0.8387539 |
|  | ARI | 0.2167431 | 0.7731401 | 0.5169885 |
|  | FM | 0.4135288 | 0.810128 | 0.6259309 |
|  | Jaccard | 0.2606435 | 0.6800513 | 0.4431576 |
| DeepImpute | Rand | 0.6785642 | 0.901179 | 0.7789353 |
|  | ARI | 0.2360079 | 0.6157208 | 0.3206709 |
|  | FM | 0.461937 | 0.6740766 | 0.4627405 |
|  | Jaccard | 0.2954387 | 0.5082101 | 0.2893786 |

*The data is corresponding to Figure 8.*
